# Gray wolf optimization-extreme learning machine approach for diabetic retinopathy detection

**DOI:** 10.3389/fpubh.2022.925901

**Published:** 2022-08-01

**Authors:** Musatafa Abbas Abbood Albadr, Masri Ayob, Sabrina Tiun, Fahad Taha AL-Dhief, Mohammad Kamrul Hasan

**Affiliations:** ^1^Center for Artificial Intelligence Technology (CAIT), Faculty of Information Science and Technology, Universiti Kebangsaan Malaysia, Bangi, Malaysia; ^2^Department of Communication Engineering, School of Electrical Engineering, Universiti Teknologi Malaysia (UTM) Johor, Bahru, Malaysia; ^3^Faculty of Information Science and Technology, Center for Cyber Security, Universiti Kebangsaan Malaysia, Bangi, Malaysia

**Keywords:** gray wolf optimization, extreme learning machine, Histogram of Oriented Gradients, Principal Component Analysis, Diabetic Retinopathy

## Abstract

Many works have employed Machine Learning (ML) techniques in the detection of Diabetic Retinopathy (DR), a disease that affects the human eye. However, the accuracy of most DR detection methods still need improvement. Gray Wolf Optimization-Extreme Learning Machine (GWO-ELM) is one of the most popular ML algorithms, and can be considered as an accurate algorithm in the process of classification, but has not been used in solving DR detection. Therefore, this work aims to apply the GWO-ELM classifier and employ one of the most popular features extractions, Histogram of Oriented Gradients-Principal Component Analysis (HOG-PCA), to increase the accuracy of DR detection system. Although the HOG-PCA has been tested in many image processing domains including medical domains, it has not yet been tested in DR. The GWO-ELM can prevent overfitting, solve multi and binary classifications problems, and it performs like a kernel-based Support Vector Machine with a Neural Network structure, whilst the HOG-PCA has the ability to extract the most relevant features with low dimensionality. Therefore, the combination of the GWO-ELM classifier and HOG-PCA features might produce an effective technique for DR classification and features extraction. The proposed GWO-ELM is evaluated based on two different datasets, namely APTOS-2019 and Indian Diabetic Retinopathy Image Dataset (IDRiD), in both binary and multi-class classification. The experiment results have shown an excellent performance of the proposed GWO-ELM model where it achieved an accuracy of 96.21% for multi-class and 99.47% for binary using APTOS-2019 dataset as well as 96.15% for multi-class and 99.04% for binary using IDRiD dataset. This demonstrates that the combination of the GWO-ELM and HOG-PCA is an effective classifier for detecting DR and might be applicable in solving other image data types.

## Introduction

Diabetic Retinopathy (DR) is a condition of the eye that can cause blindness and vision loss in individuals who have diabetes. Regular examination of the eyes is essential for early retinopathy detection in order to decrease the blindness and vision loss caused by DR (1). The core objective of the DR examination is to reveal whether further treatments are required or not (2). Therefore, a robust and accurate retinal examination system is desired to help the screeners to classify the retinal images effectively as well as with high confidence.

Nowadays, Artificial Intelligence (AI) and Machine Learning (ML) techniques are playing significant roles in aiding medical experts with early illness diagnosis (3–6). Therefore, recently, research has been conducted using various AI and ML techniques in order to automatically detect the DR using images (7–9). One of the well-known feature extraction techniques is Histogram of Oriented Gradients (HOG) and has been widely utilized in many image processing fields, including medical fields (10–12). Moreover, the Principal Component Analysis (PCA) is considered one of the most recognized dimensionality reduction techniques (13), where it condenses most of the information in the database into a small dimensions' number. In addition, recently, the Gray Wolf Optimization-Extreme Learning Machine (GWO-ELM) has been considered one of the most popular ML algorithms (14). Therefore, the major aims of this study are as follows:

- Propose a new DR detection approach based on a GWO-ELM classifier and Histogram of Oriented Gradients- Principal Component Analysis (HOG-PCA) features using image data.
- Test the proposed approach based on two different DR image datasets [i.e., APTOS-2019 and Indian Diabetic Retinopathy Image Dataset (IDRiD)] in both binary and multi-class classifications.
- The NN, SVM, Random Forest (RF), and basic ELM classifiers are also implemented in both binary and multi-class classifications using APTOS-2019 and IDRiD datasets.
- Evaluate the performance of the proposed DR detection approach based on several evaluation measures such as accuracy, recall, precision, specificity, F-measure, G-mean, and Matthews Correlation Coefficient (MCC).
- Compare the proposed DR approach against the most recent studies that have used the same datasets in terms of accuracy for the binary and multi-class classifications.

This research is organized as follows: Section 2 presents the related work of this study. Section 3 provides a deep explanation and description of the materials and proposed method. Section 4 discusses the experiments and their outcomes. Section 5 presents the conclusion of this research as well as recommendations for future research.

## Related work

The authors in Sridhar et al. (15) have proposed an automatic system for detecting DR by using Convolutional Neural Network (CNN). The proposed system was tested based on binary classification and used an image dataset that is available on the Kaggle website. The experiments' outcomes have shown that the highest performance of their proposed CNN was achieved with an accuracy of 86%. However, they have tested the proposed system based on binary classification only and ignored the multi-class classification. In addition, the accuracy rate is still not encouraging and needs more enhancement.

Another attempt has been conducted in Gangwar and Ravi (16). They proposed a hybrid architecture of inception-ResNet-v2 and custom CNN layers for the detection of DR. The proposed model was evaluated based on the multi-class classification using APTOS-19 and Messidor-1 dataset. Results showed that the highest accuracy achieved by the proposed model is 72.33% on the Messidor-1 dataset and 82.18% on the APTOS-19 dataset.

One of the most popular ML algorithms is Extreme Learning Machine (ELM); ELM is a single hidden layer feed-forward neural network that consists of three layers (i.e., input, hidden, and output layers) (17, 18). The neurons of the input layer are connected to the neurons of the hidden layer by randomly generated input weights and biases. The neurons of the hidden layer are connected to the neurons of the output layer by output weights. The output weights are calculated based on discovering the least-squares solution (19, 20). ELM is preferred by researchers as it is superior to traditional Support Vector Machine (SVM) and Back Propagation Neural Network (BPNN) (21, 22) specifically in: (1) preventing overfitting, (2) its implementation on multi and binary classifications, and (3) its similar kernel-based capability SVM and working with a Neural Network (NN) structure. These factors make the ELM more efficient in accomplishing a better learning performance. Therefore, some researchers have implemented the ELM algorithm in DR detection. For example, the authors in Asha and Karpagavalli (23) have proposed a DR detection system. The system is based on combining several extracted features such as standard deviation, mean, edge strength, and centroid as well as using the ELM classifier. The system was evaluated based on a binary classification by using the DIARETDB1 dataset which contains 100 images in total. The experiment results showed that the performance of the ELM outperformed both Naive Bayes (NB) and Multilayer Perceptron (MLP) with the highest achieved accuracy reaching up to 90%.

In addition, the authors in Zhang and An (24) have proposed an automatic DR detection system. The proposed system uses two features extraction methods (i.e., lesion detection and anatomical part recognition) and Kernel Extreme Learning Machine (KELM) with an active learning technique for the classification process. The evaluation of the proposed system has been conducted based on binary classification using the Messidor dataset. The results have shown that the highest performance of the proposed system was achieved with an accuracy of 88.60%.

Further, Punithavathi and Kumar (25) used four different feature extraction techniques (i.e., mean, standard deviation, entropy, and third momentum) and the ELM classifier in order to detect DR. The proposed DR detection system was tested based on a multi-class classification problem using the DIARETDB0 dataset with four different classes. The outcomes of the experiments have proved the superiority of the ELM performance over both BPNN and SVM with the highest achieved accuracy of 95.40%.

Additionally, Deepa et al. (26) proposed a DR detection system that has three different phases. The first phase is to use several micro-macro feature extraction algorithms. The second phase is to apply the Principal Component Analysis (PCA) on the extracted features in order to reduce the dimensionality. Finally, the third phase is to implement the KELM on the extracted features with low dimensions for classification purposes. The proposed system was tested based on a dataset with four classes, which has been collected by the department of medical retina at Bharath hospital in Kottayam. The outcomes of the experiments have demonstrated that the highest achieved accuracy rate of the proposed system reached up to 93.20%.

Although (23–26) showed that the ELM and KELM outperformed their comparatives, these studies have ignored the fact that the random generated input weights and biases of the ELM and KELM need to be optimized. In other words, there is no guarantee that the trained ELM/KELM is the best for carrying out the classification. This drawback can be resolved by integrating the ELM/KELM with an optimisation approach to achieve the optimal input weights and hidden layer biases that guarantee the best ELM/KELM performance (27). Therefore, one of the most popular improvements of the ELM is the Gray Wolf Optimization-Extreme Learning Machine (GWO-ELM), where the GWO is integrated into ELM in order to obtain the best input weights and biases (14). GWO was established by studying the hunting behavior of gray wolves (28). It has a simple concept with easy implementation, requiring very few coding lines, allowing many to leverage from it. In comparison to other evolutionary algorithms, GWO is highly robust in regulating parameters with greater computational efficacy (29, 30). The effectiveness of this integration (GWO-ELM) has been proven in many domains including breast cancer diagnosis (31), poison diagnosis (32), lung cancer classification (33), identification of cardiovascular disease (34), electricity load projections (35), bankruptcy predictions (36), and paraquat poisoned patients diagnosis (37). However, to the best of our knowledge, no research has used the GWO-ELM classifier in the detection of DR. Therefore, this study aims to employ the GWO-ELM classifier for detecting DR. Table 1 provides a summary of the previous DR detection works using ML and deep learning techniques.

**Table 1:** Illustrates the previous works of DR detection using ML and deep learning techniques.

**References**	**Dataset**	**Classification mode**	**Classifier**	**Results**	**Disadvantages**
Sridhar et al. (15)	Kaggle dataset	Binary classification	CNN	86% Accuracy	• The proposed system tested based on binary classification only and ignored the multi-class classification. • The accuracy rate is still not encouraging and needs more enhancement.
Gangwar and Ravi. (16)	APTOS-19 and Messidor-1	Multi-class classification	Hybrid CNN	72.33% Accuracy on the Messidor-1 dataset and 82.18% accuracy on the APTOS-19 dataset.	• The evaluation of both systems considered only the multi-class classification and ignored the binary classification. • The accuracies of both systems are still not promising and need more improvement.
Reddy et al. (38)	Messidor	Multi-class classification	SVM	69.09% Accuracy	
Asha and Karpagavalli. (23)	DIARETDB1	Binary classification	ELM	90% Accuracy	• The proposed system tested based on binary classification only and ignored the multi-class classification. • The accuracy rates are still not encouraging and need more enhancement. • These studies have ignored the fact that the random generated input weights and biases of the ELM and KELM need to be optimized.
Zhang and An (24)	Messidor	Binary classification	KELM	88.60% Accuracy	
Punithavathi and Kumar (25)	DIARETDB0	Multi-class classification	ELM	95.40% Accuracy	• The evaluation of both systems considered only the multi-class classification and ignored the binary classification. • The accuracy rates are still not encouraging and need more enhancement. • These studies have ignored the fact that the random generated input weights and biases of the ELM and KELM need to be optimized.
Deepa et al. (26)	4 classes dataset	Multi-class classification	KELM	93.20%	

## Materials and proposed method

**Figure 1:**
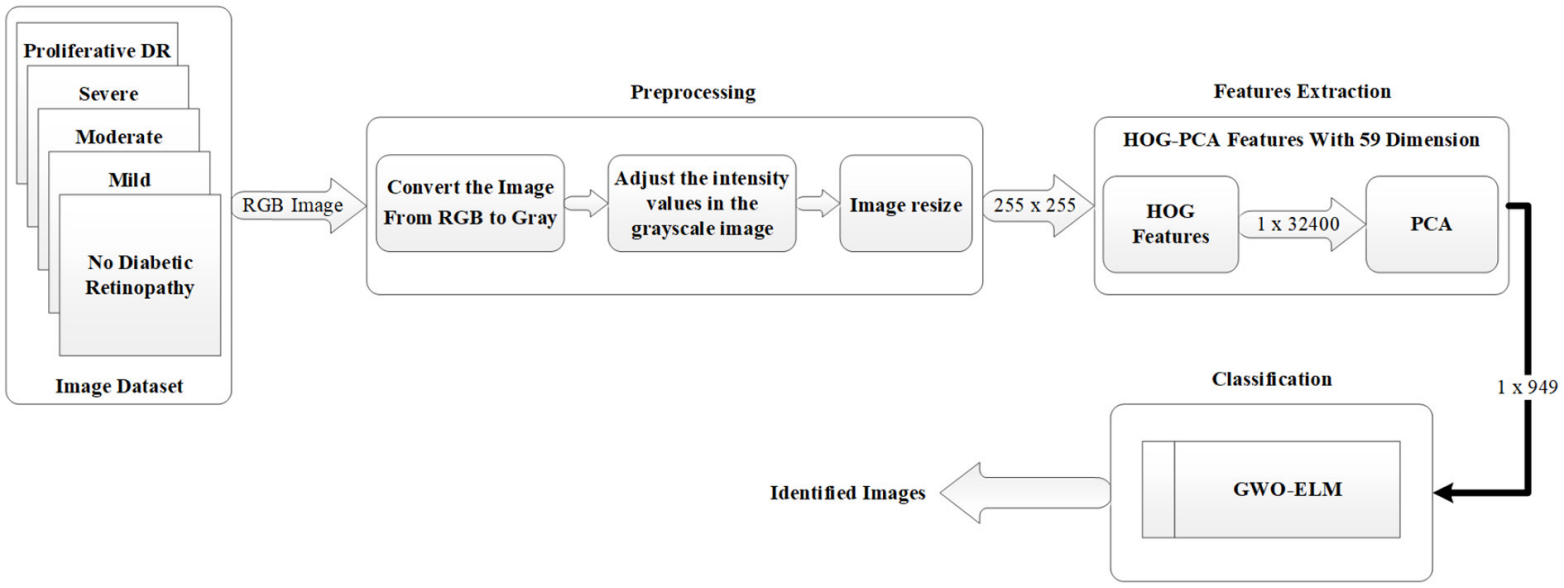
Block diagram of the proposed DR detection approach.

The general diagram of the proposed work using the GWO-ELM method is demonstrated in Figure 1. The diagram consists of various stages which will be used to create the DR detection approach based on images. The first stage refers to the image dataset that contains five categorizations (i.e., no DR, mild, moderate, severe, and proliferative DR). While, in the second stage, the pre-processing operation will be used in order to prepare the images for the next stage, which is the features extraction stage. In addition, in the third stage, the HOG-PCA method will be utilized in order to extract the needed features from images. Lastly, in the fourth stage, the HOG-PCA extracted features will be fed into the GWO-ELM classifier in order to detect DR based on images. These fourth stages of the proposed DR detection approach will be deliberated as sub-sections, respectively.

### Image dataset

In this study, two different datasets will be used in order to evaluate the proposed DR detection approach. The first dataset is APTOS-2019 while the second dataset is IDRiD. The description of both datasets APTOS-2019 and IDRiD are provided as follow:

- APTOS-2019 Dataset has been provided by an Indian hospital, Aravind Eye Hospital. The APTOS-2019 dataset is available online in Hospital (39). In this study, the dataset consists of five main classes, which are no DR, mild, moderate, severe, and proliferative DR, and each class contains 190 images. Thus, 950 is the total number of images in the whole dataset. In this study, 80% of the dataset, which equals 760 images, were used for training purposes, whilst 20% of the dataset, which equals 190 images, were used for testing purposes. In other words, 152 images from each class were used for training purposes whilst the remaining 38 images were used for testing purposes. The description of the APTOS-2019 dataset which is used in this study is provided in Table 2.
- IDRiD is a DR image dataset that is available online at (40). The IDRiD dataset consists of five main classes, which are no DR, mild, moderate, severe, and proliferative DR. In addition, the IDRiD dataset has a total number of images equal to 516 and each class contains a different number of images. In this study, 80% of the dataset that equals 412 images were used for training purposes, whilst the remaining 20% of the dataset which equals 104 images were used for testing purposes. The description of the IDRiD dataset which is used in this study is provided in Table 3.

**Table 2:** The description of the APTOS-2019 dataset.

**Class**	**Number**	**Samples of the dataset**			**Class label**
	**of image**				
No DR	190	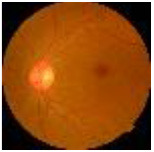	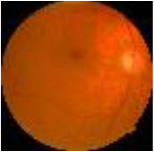	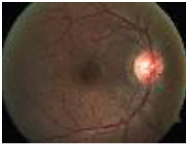	1
Mild	190	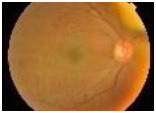	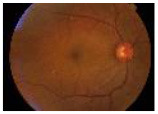	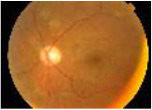	2
Moderate	190	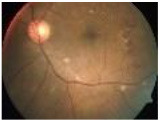	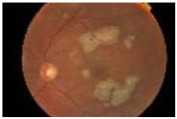	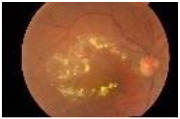	3
Severe	190	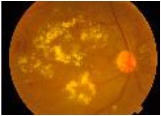	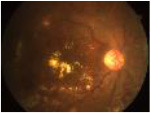	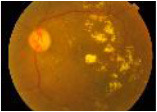	4
Proliferative DR (PDR)	190	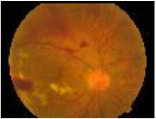	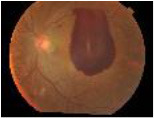	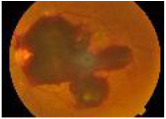	5

**Table 3:** The description of the IDRiD dataset.

**Class**	**Number**	**Samples of the dataset**			**Class label**
	**of image**				
No DR	168	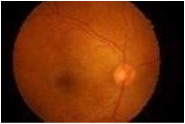	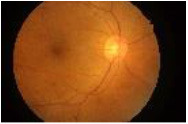	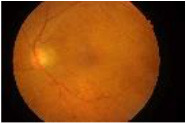	1
Mild	25	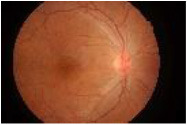	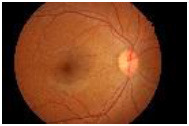	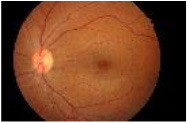	2
Moderate	168	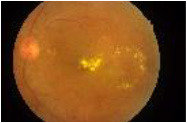	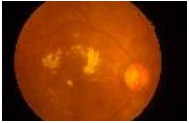	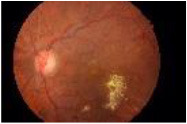	3
Severe	93	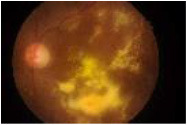	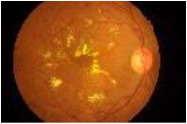	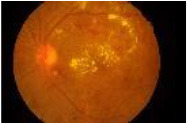	4
Proliferative DR (PDR)	62	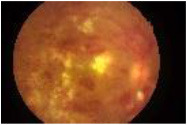	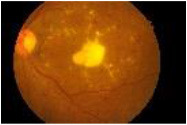	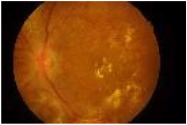	5

### Pre-processing

**Figure 2:**
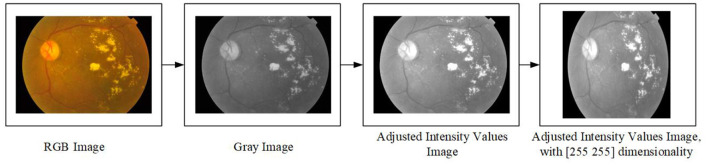
The pre-processing steps.

This section discusses the pre-processing of this study, which consists of four steps. The first step is to read the RGB image that will be as an array with three dimensions. The second step is to convert the image from RGB to Grayscale, which will lead to making it an array with two dimensions. The third step is to adjust the intensity values in the grayscale image which leads to an increase in the contrast of the output image. Finally, the fourth step is to re-size the dimensionality of the image to (255 × 255) which will be as an input into the features extraction approach. Figure 2 depicts an example of the pre-processing steps which are used in this study.

### Features extraction

In this work, the required features were calculated in two steps. The first step is to use the output of the pre-processing as an input into the Histogram of Oriented Gradients (HOG) features extraction technique, which begins after the pre-processing phase. HOG is considered as one of the most popular features extraction techniques that has been widely utilized in many image processing fields, including medical fields (10–12). The output of the HOG features extraction approach is a vector with the dimensionality of (1 × 32,400) per image, and (950 × 32,400) and (516 × 32,400) for whole APTOS-2019 and IDRiD dataset, respectively.

**Figure 3:**
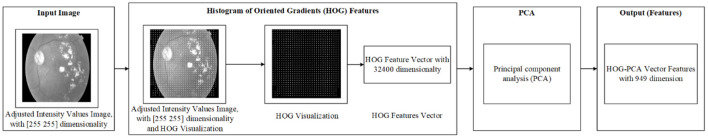
Steps of the features extraction.

Whilst the second stage is to reduce the dimensionality of HOG features using Principal Component Analysis (PCA). PCA is considered one of the most recognized dimensionality reduction techniques (13), where it condenses most of the information in the database into a small dimensions' number. The aim of that is to reduce the high dimensionality of the HOG features from (950 × 32,400) to (950 × 949) for whole APTOS-2019 dataset and from (516 × 32,400) to (516 × 515) for whole IDRiD dataset. This enables the issue of limited resources (i.e., requiring a large memory space) to be overcome. Literature has addressed the issue that the required memory space is affected by the dimensionality of the features (i.e., number of features). In other words, the higher dimensionality requires a large memory space (41–43). The final output of the features extraction is the HOG-PCA features with (950 × 949) dimensionality for whole APTOS-2019 dataset and (516 × 515) for whole IDRiD dataset, both of which will be used as inputs into the classification step. Figure 3 demonstrates the steps of the features extraction in more detail. Further, Table 4 demonstrates the dimensionality of the features extraction steps for a single image and whole dataset images.

**Table 4:** Elaborate the features extraction step dimensionality for single image and whole dataset images.

**APTOS-2019 Dataset**		
**Features Extraction**	**Dimensionality of a single image**	**Dimensionality of the whole dataset**
First Step: HOG Features	(1 x 32400)	(950 x 23400)
Second Step: HOG-PCA Features	(1 x 949)	(950 x 949)
**IDRiD Dataset**		
**Features extraction**	**Dimensionality of a single image**	**Dimensionality of the whole dataset**
First Step: HOG Features	(1 x 32400)	(516 x 32400)
Second Step: HOG-PCA Features	(1 x 515)	(516 x 515)

### Classification

This section provides a deep explanation of both GWO and GWO-ELM approaches separately. The explanation of the GWO approach is delivered in Section 2.4.1, while the explanation of the GWO-ELM approach is presented in Section 2.4.2.

#### Gray wolf optimization

In recent years, GWO has emerged as a prominent new nature-based metaheuristic algorithm and population-oriented metaheuristic (30). GWO is based on the natural behaviors of the gray wolf (28). The algorithm fundamentally simulates the wolf's social behavior and hunting mechanisms. In GWO, the wolves (search agents) are classified as alpha (α), beta (β), delta (δ), and omega (ω). α is the fittest wolf or the best solution. β and δ each denote the second and third best wolves. Meanwhile, ω denotes the other wolves in the population. Finding the prey (process of optimization) is spearheaded by δ, β, and α whilst the wolves (ω) are the followers. When surrounding the prey, wolves inform about their positions based on δ, β, or α using the following equations (28):


(1) $$\begin{array}{r} D=|C\cdot X_{p}\left(it\right)-X\left(it\right)| \end{array}$$

and


(2) $$\begin{array}{r} X\left(it+1\right)=X_{p}\left(it\right)-A\cdot D \end{array}$$

Where, *it* denotes the present iteration number. *X*_*p*_ *(it)* denotes the present position of the prey. *X (it)* denotes the wolf's present position. *D* denotes the distance between the prey and wolf. Below are the mathematical formulas for coefficient vectors (A and C):


(3) $$\begin{array}{r} A=2a\cdot r_{1}-a \end{array}$$

and


(4) $$\begin{array}{r} C=2\cdot r_{2} \end{array}$$

Where *r*_1_ and *r*_2_ are the two vectors that are randomly generated between 1 and 0. “*a*” denotes linear decrement from 2 to 0 as the iterations number increase. The simulation of the wolves' hunting behaviors results in the saving of the first three top values as α, β, and δ. Below is the formula for updating the position of the gray wolf population:


(5) $$\{\begin{array}{c} D_{\alpha }=|C_{1}\cdot X_{\alpha }-X|\\ D_{\beta }=|C_{2}\cdot X_{\beta }-X|\\ D_{\delta }=|C_{3}\cdot X_{\delta }-X| \end{array}$$

(6) $$\left\{ \begin{array}{c} X_{1}=X_{\alpha }(it)-A_{1}\cdot D_{\alpha }\\ X_{2}=X_{\beta }(it)-A_{2}\cdot D_{\beta }\\ X_{3}=X_{\delta }(it)-A_{3}\cdot D_{\delta } \end{array}\right.$$

and


(7) $$\begin{array}{r} X\left(t+1\right)=\frac{X_{1}+X_{2}+X_{3}}{3} \end{array}$$

Where *X*_α_, *X*_β_, and *X*_δ_ denote the positions of α, β, and δ, respectively. *X* denotes the current wolf position. *C*_1_, *C*_2_, and *C*_3_ are vectors that are randomly generated based on Equation (4). Equation (5) is used to calculate the estimated distances among the current wolf and α, β, and δ, whilst Equations (6) and (7) are used to determine the current wolf's final position. *A*_1_, *A*_2_, and *A*_3_ are vectors that randomly generated using Equation (3). *it* represents the iterations number.

**Figure 4:**
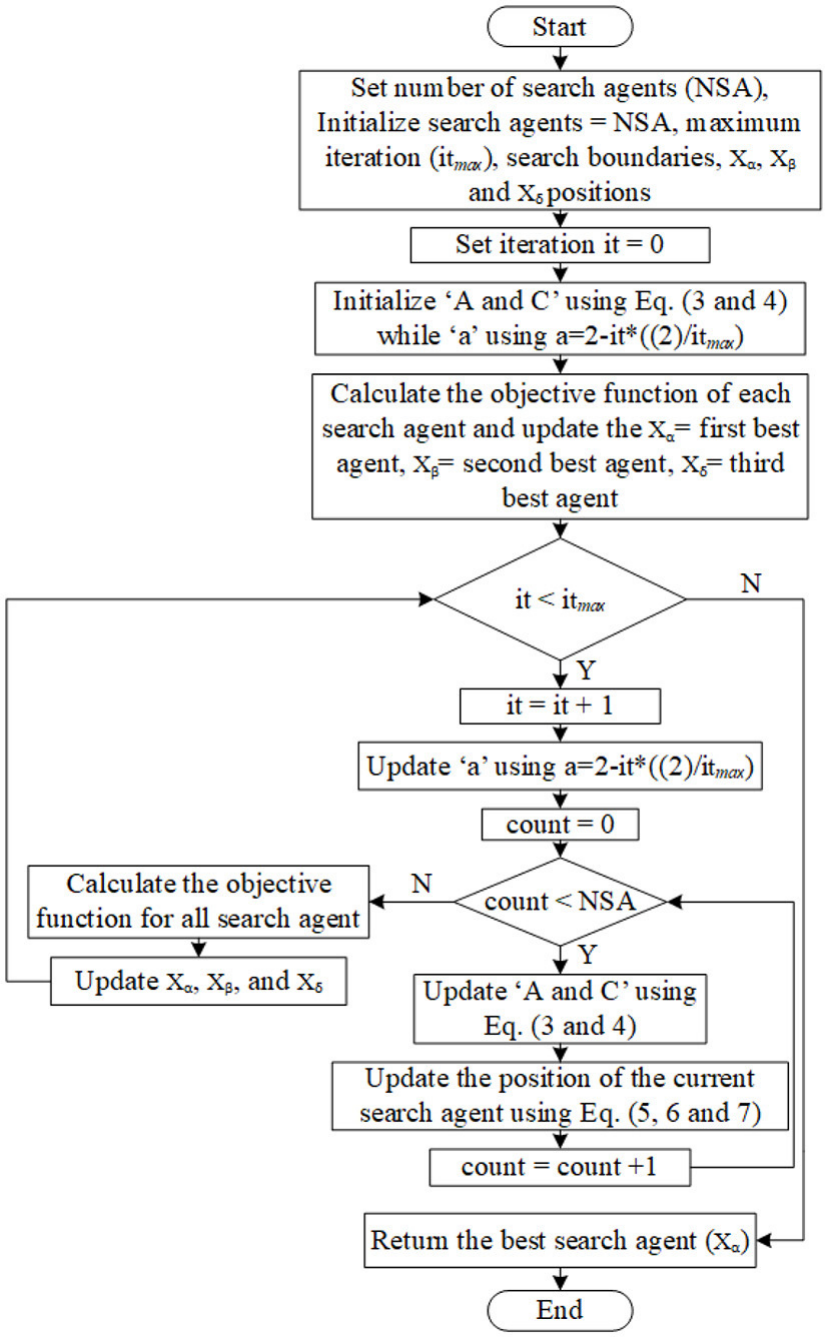
Flowchart of the GWO algorithm.

This updating mechanism facilitates the omega wolves in reaching new stochastic places (presumed to be nearer to the prey) in the circle delineated by the leading wolves' positions. GWO is distinguished by its strategy in managing the explorations and exploitations in the search process. With a decrease from 2 to 0 during the iterations, the algorithm progressively moves on from underlining the process of exploration to the process of exploitation (30). Figure 4 shows the GWO algorithm flowchart. Below are the general processing steps of the GWO algorithm (28):

(a) Parameters of the gray wolf, such as population size or the number of search agents (NSA), are initialized. For the following steps, the search agent term refers to a wolf, position of each wolf (search agent), maximum number of iteration (*it*_*max*_), and upper and lower bound of search.
(b) Set the iteration counter it = 0.
(c) Initialize the coefficient vectors “*A*, and *C*” using Equations (3 and 4) while the initialization of “a”, which is the linear decrements from 2 to 0 as the iterations number increase, uses *a* = 2-it^*^((2)/ it_*max*_*)*.
(d) Calculate the fitness for all search agents and set the first three best search agents as *X*_α_, *X*_β_, and *X*_δ_ where *X*_α_ denotes the first best search agent whilst *X*_β_ denotes the second best search agent, and *X*_δ_ denotes the third best search agent.
(e) Increase the iteration counter *it* = *it* + *1*.
(f) Update “*A*, and *C*” using Equations (3 and 4) while “a” using *a*=2-it^*^((2)/ it_*max*_*)*.
(g) Update the position of all current search agents using Equations (5 and 6).
(h) Recalculate the fitness for all search agents.
If any better search agent is found, then update the best agents *X*_α_, X_β_, X_δ_.
(j) Repeat steps from “e” if the stopping criteria are not satisfied.
(k) The best-calculated optimum (best search agent) will be returned as *X*_α_.

#### GWO-ELM

**Figure 5:**
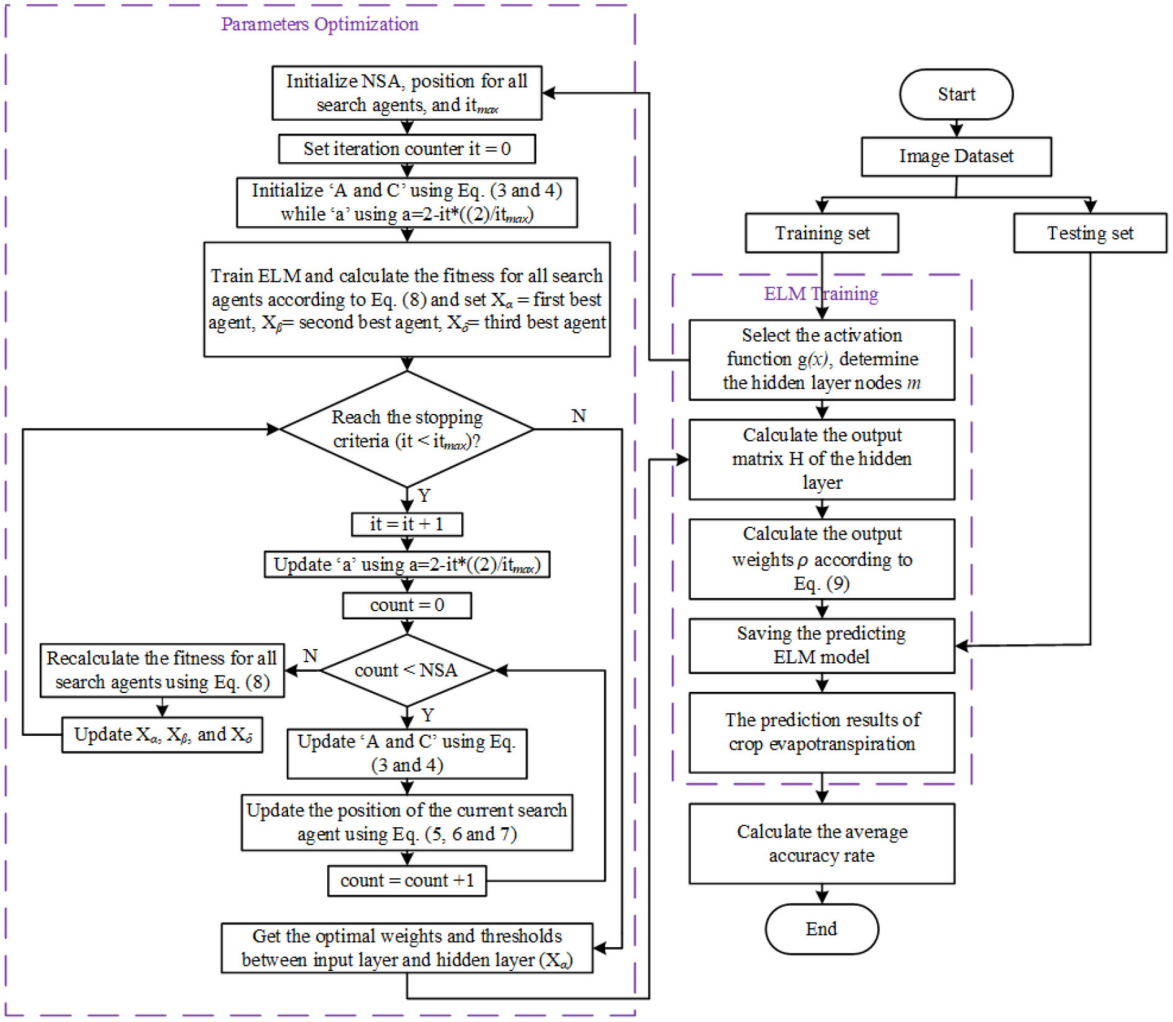
GWO-ELM algorithm flowchart.

The GWO-ELM follows the GWO concept in Mirjalili et al. (28). It adjusts the input weight values and the biases of the hidden nodes by updating the GWO parameters toward achieving greater accuracy. The GWO-ELM steps are presented below while the flowchart is illustrated in Figure 5. Table 5 shows the ELM-GWO parameter settings.

**Table 5:** The parameters settings for the ELM and GWO.

**ELM**		**GWO**	
**Parameter**	**Value**	**Parameter**	**Value**
*AS*	assemble of the biases and input weights	Population (wolves or search agents)	Consists of the position of all search agents
ρ	Output-weights matrix	Position	Start stochastically generated within the range of [-1, 1] for the input-weights and [0, 1] for the biases
Input-weights (*w*)	−1 to 1	Population size or number of search agents (NSA)	50
Bias values (*b*)	0 to 1	r_1_ and r_2_	Stochastically generated with the range of [0, 1]
Input-nodes number (*n*)	Input attributes	Number of iterations it*_*max*_*	100
Hidden-nodes number (*L*)	[100–300]; with a 25 increment step	C_1_, C_2_, and C_3_	Randomly generated vectors based on Equation (4)
Output neurones number (*m*)	Number of classes	A_1_, A_2_, and A_3_	Randomly generated vectors using Equation (3)
Activation function	Sigmoid	X_α_	Best position of all search agents.

Let *N* be the number of training samples and *(X*_*j*_*, t*_*j*_*)* refer to a single sample of the training samples.,

Where:

*X*_*j*_ is the input extracted from HOG-PCA features where *X*_*j*_ = *[x*_*j*1_*, x*_*j*2_*, …, x*_*jn*_*]*^*T*^ ∈ *R*^*n*^,

*t*_*j*_ is the expected output (true value) where *t*_*j*_ = *[t*_*j*1_*, t*_*j*2_*, …, t*_*jm*_*]*^*T*^ ∈ *R*^*m*^.

**Step 1:** Random initialization of the gray wolf population (position of all search agents) within the range of [-1, 1] for the values of the input weights, and [0, 1] for the hidden nodes' bias. Ascertaining the initial GWO parameters entails: 1) the population size or number of search agents (NSA), 2) the maximum number of iterations (*it*_*max*_), and 3) the iteration counter *it* = 0. Each wolf (search agent) in the population is reshaped using the following form:

$$\begin{array}{l} SA_{i}=\left[\begin{array}{cccccccc} w_{11,} & w_{12,} & \ldots & w_{1n,} & w_{21,} & w_{22,} & \ldots \\ w_{2n,} & w_{L1,} & w_{L2,} & \ldots & w_{Ln,} & b_{1,} & \ldots & b_{L} \end{array}\right] \end{array}$$

Where:
*w*_*ij*_ = value of input-weights which connect between the *i*_*th*_ hidden node and *j*_*th*_ input node, *w*_*ij*_∈ [−1, 1].
*b*_*i*_ = *i*_*th*_ hidden node's bias, *b*_*i*_ ∈ [0, 1].
*n* = number of the input-nodes.
*L* = number of the hidden nodes.
*L* × *(1*+*n)* denotes the dimension of the search agent, which therefore requires optimization of its parameters.
**Step 2:** Initialization of the coefficient vectors ‘*A*, and *C*' using Equations (3 and 4) while the initialization of the ‘a' which is the linear decrements from 2 to 0 as the iterations number increase, using a = 2-it^*^((2)/ it_max_).
**Step 3:** Division of the dataset into training and testing sets
Set the hidden layer nodes as *m*, and choose a suitable activation function *g(x)* for ELM;

(8) $$\begin{array}{r} f\left(X\right)=\sqrt{\frac{\overset{N}{\underset{j=1}{\sum }}||\overset{L}{\underset{i=1}{\sum }}\rho_{i}g\left(w_{i}x_{j}+\;b_{i}\right)-t_{j}{\left|\right|}_{2}^{2}}{N}} \end{array}$$

Where:
ρ = output weight matrix;
*t*_*j*_ = true value; and
*N* = number of training samples.
Where:

(9) $$\begin{array}{r} \rho =H^{†}T \end{array}$$

(10) $$\begin{array}{r} H={\left[\begin{array}{ccc} g\left(w_{1}.X_{1}+b_{1}\right) & \cdots & g\left(w_{L}.X_{1}+b_{L}\right)\\ \vdots & \ldots & \vdots \\ g\left(w_{1}.X_{N}+b_{1}\right) & \cdots & g\left(w_{L}.X_{N}+b_{L}\right) \end{array}\right]}_{N\times L} \end{array}$$

$$\rho ={\left[\begin{array}{c} \rho_{1}{}^{T}\\ \vdots \\ \rho_{L}{}^{T} \end{array}\right]}_{L\times m}and\;T\;={\left[\begin{array}{c} t_{1}{}^{T}\\ \vdots \\ t_{N}{}^{T} \end{array}\right]}_{N\times \;m}$$

*H* in Equation (10) is the hidden layer output matrix of the ELM network; in *H*, the *i*_*th*_ column is indicated to the *i*_*th*_ hidden layer neuron on the input neurons. While the *H*^†^ is the Moore–Penrose generalized inverse of *H*. The activation function *g* is infinitely distinguishable when the desired number of hidden neurons is *L* ≤ *N*.
**Step 4:** Train the ELM and evaluate the fitness value of each search agent according to the accuracy of the classification.
**Step 5:** Based on the fitness values of each search agent, set the first three best search agents as *X*_α_, *X*_β_, and *X*_δ_, where *X*_α_ denotes the first best search agent whilst *X*_β_ denotes the second best search agent, and *X*_δ_ denotes the third best search agent.
**Step 6:** Increase the iteration counter *it* = *it* + *1*.
**Step 7:** Update ‘*A*, and *C*' using Equations (3 and 4) while ‘*a*' using *a* = 2-it^*^((2)/ it_*max*_*)*.
**Step 8:** Update the position of all current search agents using Equations (5–7).
**Step 9:** Recalculate the fitness for all search agents using Equation (8).
**Step 10:** If any better search agent is found, then update the best agents *X*_α_, *X*_β_, *X*_δ_.
**Step 11:** Repeat steps from step 6 if the stopping criteria are not satisfied, or else save the optimal weights and thresholds between input layers and hidden layers (*X*_α_).
**Step 12:** The results of GWO are utilized as input-weights and hidden-layer biases of the ELM, calculating the hidden layer output matrix (*H*) *via* the activation function *g(x)*;
**Step 13:** Calculate the output-weights (ρ) according to Equation (9) and save the forecasting ELM model for testing.

## Experiments and results

The proposed GWO-ELM approach was utilized in both binary and multi-class classification experiments with a hidden neurons number in a range of [100–300] and increment steps of 25. In the multi-class classification experiments, we have used both APTOS-2019 and IDRiD datasets in order to classify five different classes, namely no DR, mild, moderate, severe, and proliferative DR. In the binary classification experiments, we have used both APTOS-2019 and IDRiD datasets in order to classify two different classes (i.e., no DR and DR). The class of DR was obtained by combining mild, moderate, severe, and proliferative DR classes. Hence, the total number of both binary and multi-class classification experiments for the GWO-ELM approach is 36, and each experiment has 100 iterations. All the experiments have been applied based on using 80% of the dataset as a training dataset and the remaining 20% as a testing dataset. In addition, it is worth mentioning that all the experiments have been implemented in MATLAB R2019a programming language over a PC Core i7 of 3.20 GHz with 16 GB RAM and SSD 1 TB (Windows 10).

In this study, numerous evaluation measurements were utilized to evaluate the proposed approach GWO-ELM. The evaluation measurements rely on the ground truth, which entails the application of the model to expect the answer on the evaluation dataset followed by a comparison between the predicted target and the actual answer. The evaluation measurements have been used in order to evaluate the proposed GWO-ELM approach regarding True Positive (TP), True Negative (TN), False Positive (FP), False Negative (FN), recall, accuracy, specificity, G-mean, precision, F-measure, and MCC. Equations (11–17) (44–46) depict these evaluation measurements.


(11) $$\begin{array}{r} accuracy=\frac{TP+TN}{TP+TN+FN+FP} \end{array}$$

(12) $$\begin{array}{r} precision=\frac{TP}{TP+FP} \end{array}$$

(13) $$\begin{array}{r} recall=\frac{TP}{TP+FN} \end{array}$$

(14) $$\begin{array}{r} F-Measure=\frac{\left(2\;\times \;precision\;\times \;recall\right)}{\left(precision\;+\;recall\right)} \end{array}$$

(15) $$\begin{array}{r} G-Mean=\sqrt[{2}]{recall\times precision} \end{array}$$

(16) $$\begin{array}{r} Specificity=\frac{TN}{TN+FP} \end{array}$$

(17) $$\begin{array}{c} MCC=\frac{TP\;\times \;\;TN-FP\;\times FN}{\sqrt{\left(TP+FP)\;\times (TP+FN)\times (TN+FP)\times (TN+FN\right)}} \end{array}$$

**Figure 6:**
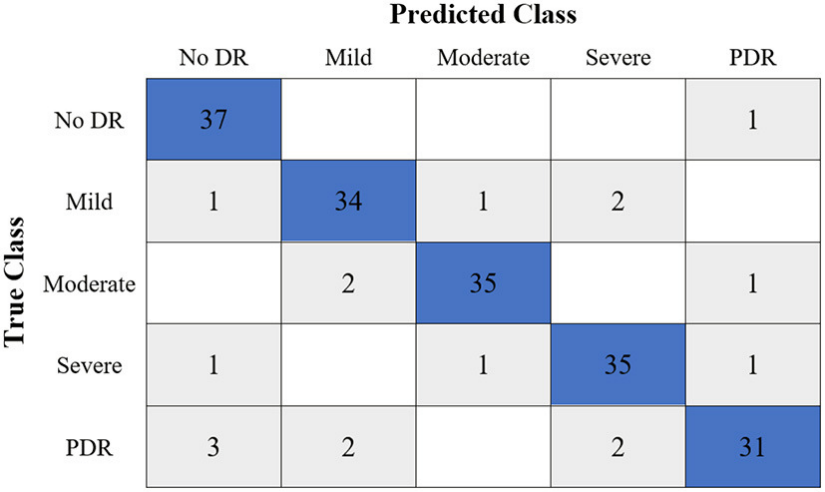
The confusion matrix of the highest multi-class classification outcome for the GWO-ELM approach using the APTOS-2019 dataset.

**Figure 7:**
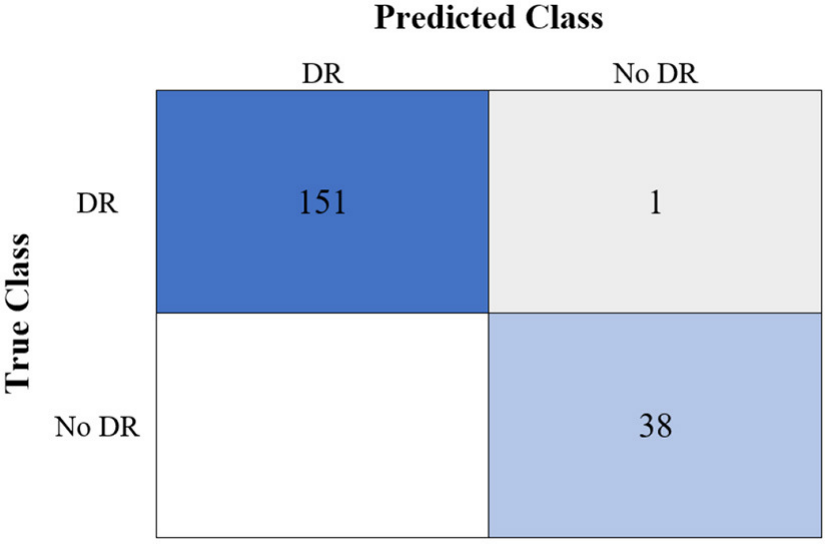
The confusion matrix of the highest binary classification outcome for the GWO-ELM approach using the APTOS-2019 dataset.

**Figure 8:**
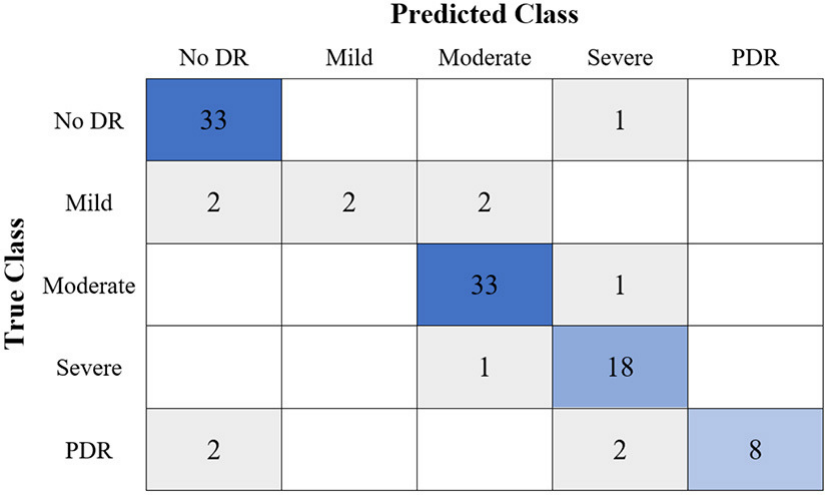
The confusion matrix of the highest multi-class classification outcome for the GWO-ELM approach using the IDRiD dataset.

**Figure 9:**
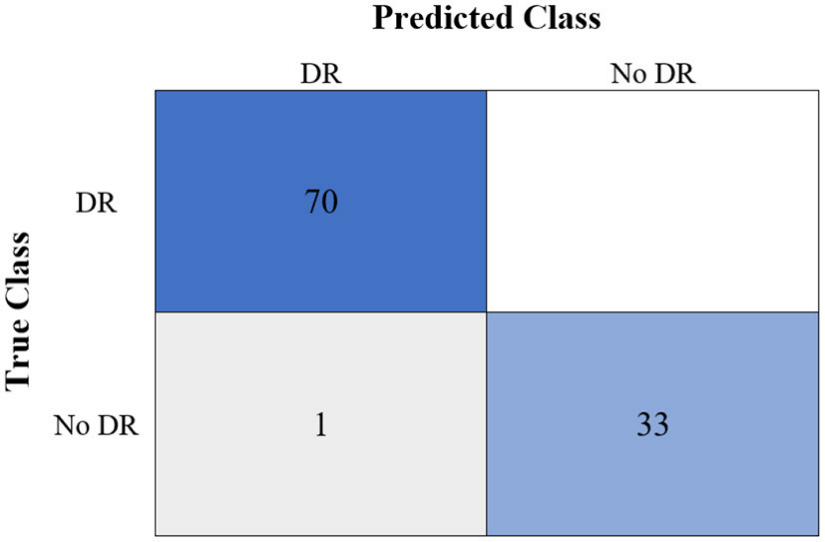
The confusion matrix of the highest binary classification outcome for the GWO-ELM approach using the IDRiD dataset.

**Figure 10:**
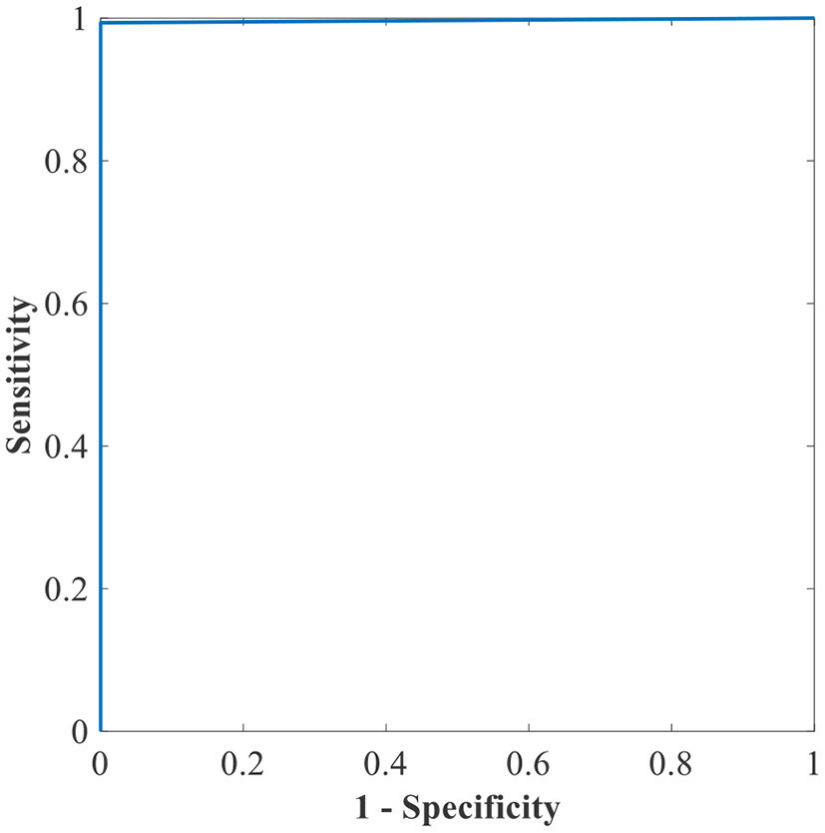
The ROC of the highest binary classification outcome for the GWO-ELM approach using the APTOS-2019 dataset.

**Figure 11:**
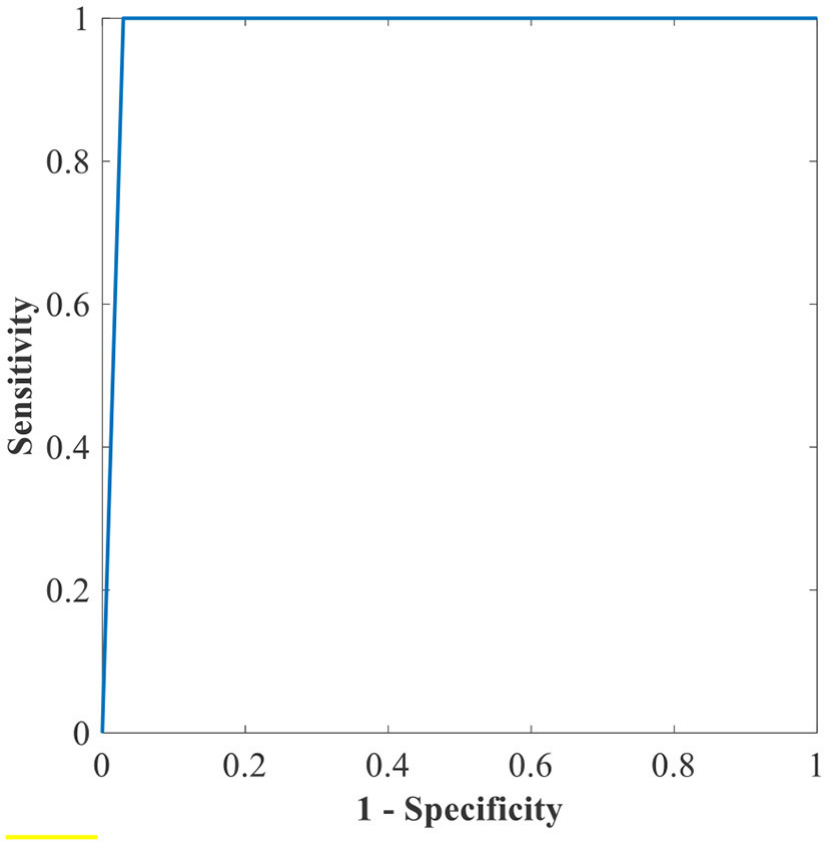
The ROC of the highest binary classification outcome for the GWO-ELM approach using the IDRiD dataset.

Table 6 shows the highest outcomes of the binary and multi-class classification experiments that have been conducted using the proposed GWO-ELM approach based on both datasets APTOS-2019 and IDRiD. Table 6 presents the evaluation outcomes of the GWO-ELM in terms of recall, accuracy, specificity, G-mean, precision, F-measure, and MCC. The highest achieved multi-class classification accuracies of the GWO-ELM approach were 96.21% and 96.15% using APTOS-2019 and IDRiD datasets, respectively. Whilst the highest achieved binary classification accuracies of the GWO-ELM approach were 99.47% using the APTOS-2019 dataset and 99.04% using the IDRiD dataset. In addition, Figures 6–10 show the confusion matrices for the highest outcomes of the binary and multi-class classification using the GWO-ELM approach based on both datasets APTOS-2019 and IDRiD. Further, Figures 10, 11 present the ROC of the best binary classification outcome for the GWO-ELM approach using the APTOS-2019 and IDRiD datasets.

**Table 6:** The highest experiment outcomes of the binary and multi-class classifications for GWO-ELM approach using APTOS-2019 and IDRiD datasets.

**APTOS-2019 dataset**							
**Number of class**	**Accuracy**	**Precision**	**Recall**	**Specificity**	**MCC**	**F-measure**	**G-mean**
5	96.21	90.53	90.53	97.63	88.16	90.53	90.53
2	99.47	99.34	100.00	97.44	98.38	99.67	99.67
**IDRiD dataset**							
**Number of class**	**Accuracy**	**Precision**	**Recall**	**Specificity**	**MCC**	**F-measure**	**G-mean**
5	96.15	90.38	90.38	97.60	87.98	90.38	90.38
2	99.04	100.00	98.59	100.00	97.82	99.29	99.29

Further, additional experiments have been implemented utilizing feedforward NN and basic ELM as classifiers and HOG-PCA features to perform binary and multi-class classification of the DR. Both classifiers NN and basic ELM were implemented in binary and multi-class classifications when varying the number of the hidden nodes in the range of [100–300] and increment steps of 25. Tables 7, 8 provide the highest binary and multi-class classification experiments outcomes of the NN and ELM classifiers using both APTOS-2019 and IDRiD datasets. The best performance of the basic ELM in multi-class classification has been obtained with an accuracy of 80.21% and 74.62% for APTOS-2019 and IDRiD datasets, respectively. While the highest performance of the basic ELM in binary classification has acquired an accuracy of 92.63% using APTOS-2019 dataset and 72.12% using IDRiD dataset. Furthermore, the best achieved multi-class classification accuracies of the NN approach were 78.53% and 72.31% using APTOS-2019 and IDRiD datasets, respectively. The highest achieved binary classification accuracies of the NN approach were 90.53% using the APTOS-2019 dataset and 71.15% using the IDRiD dataset.

**Table 7:** The highest experiment outcomes of the binary and multi-class classifications for ELM approach using APTOS-2019 and IDRiD datasets.

	**APTOS-2019 dataset**						
**Number of class**	**Accuracy**	**Precision**	**Recall**	**Specificity**	**MCC**	**F-measure**	**G-mean**
5	80.21	50.53	50.53	87.63	38.16	50.53	50.53
2	92.63	93.42	93.42	77.27	78.60	95.30	95.32
	**IDRiD dataset**						
**Number of class**	**Accuracy**	**Precision**	**Recall**	**Specificity**	**MCC**	**F-measure**	**G-mean**
5	74.62	36.54	36.54	84.13	20.67	36.54	36.54
2	72.12	85.71	75.95	60.00	32.75	80.54	80.68

**Table 8:** The highest experiments outcomes of the classification and detection for NN approach using APTOS-2019 and IDRiD datasets.

	**APTOS-2019 dataset**						
**Number of class**	**Accuracy**	**Precision**	**Recall**	**Specificity**	**MCC**	**F-measure**	**G-mean**
5	78.53	46.32	46.32	86.58	32.89	46.32	46.32
2	90.53	98.68	90.36	91.67	68.13	94.34	94.43
	**IDRiD dataset**						
**Number of class**	**Accuracy**	**Precision**	**Recall**	**Specificity**	**MCC**	**F-measure**	**G-mean**
5	72.31	30.77	30.77	82.69	13.46	30.77	30.77
2	71.15	97.14	70.83	75.00	26.04	81.93	82.95

Moreover, further experiments have been conducted utilizing SVM (linear kernel), SVM (precomputed kernel), and RF as classifiers and HOG-PCA features to perform binary and multi-class classifications of the DR. Table 9 provides the outcomes of the binary and multi-class classification experiments for the SVM (linear kernel), SVM (precomputed kernel), and RF classifiers using both APTOS-2019 and IDRiD datasets. In multi-class classification and when using APTOS-2019 dataset, the best performance of the SVM (linear) was achieved with an accuracy of 79.58% while the highest performance of the SVM (precomputed kernel) and RF classifiers was equal with an accuracy of 79.37%. Moreover, in binary classification and when using APTOS-2019 dataset, the best performance of the SVM (linear) and SVM (precomputed kernel) was equal and achieved an accuracy of 88.95% while the highest performance of RF classifier was achieved with an accuracy of 91.58%. Additionally, in multi-class classification and using IDRiD dataset, the best performance of the SVM (linear), SVM (precomputed kernel), and RF classifiers was achieved with an accuracy of 73.85, 73.08, and 74.23%, respectively. In binary classification and using IDRiD dataset, the highest performance of the SVM (linear), SVM (precomputed kernel), and RF classifiers was achieved with an accuracy of 68.27, 67.31, and 69.23%, respectively.

**Table 9:** The experiments outcomes of the binary and multi-class classification for SVM (linear kernel), SVM (precomputed kernel), and RF approaches using APTOS-2019 and IDRiD datasets.

**APTOS-2019 dataset with 5 classes**							
**Classifier**	**Accuracy**	**Precision**	**Recall**	**Specificity**	**MCC**	**F-measure**	**G-mean**
SVM (linear)	79.58	48.95	48.95	87.24	36.18	48.95	48.95
SVM (Precomputed Kernel)	79.37	48.42	48.42	87.11	35.53	48.42	48.42
RF	79.37	48.42	48.42	87.11	35.53	48.42	48.42
**APTOS-2019 dataset with 2 classes**							
**Classifier**	**Accuracy**	**Precision**	**Recall**	**Specificity**	**MCC**	**F-measure**	**G-mean**
SVM (linear)	88.95	100.00	87.86	100.00	62.69	93.54	93.73
SVM (Precomputed Kernel)	88.95	100.00	87.86	100.00	62.69	93.54	93.73
RF	91.58	100.00	90.48	100.00	72.37	95.00	95.12
**IDRiD dataset with 5 classes**							
**Classifier**	**Accuracy**	**Precision**	**Recall**	**Specificity**	**MCC**	**F-measure**	**G-mean**
SVM (linear)	73.85	34.62	34.62	83.65	18.27	34.62	34.62
SVM (Precomputed Kernel)	73.08	32.69	32.69	83.17	15.87	32.69	32.69
RF	74.23	35.58	35.58	83.89	19.47	35.58	35.58
**IDRiD dataset with 2 classes**							
**Classifier**	**Accuracy**	**Precision**	**Recall**	**Specificity**	**MCC**	**F-measure**	**G-mean**
SVM (linear)	68.27	98.57	68.32	66.67	12.48	80.70	82.06
SVM (Precomputed Kernel)	67.31	84.29	71.95	50.00	19.11	77.63	77.87
RF	69.23	100.00	68.63	100.00	20.09	81.40	82.84

The outcomes for binary and multi-class classification are shown in Tables 6–9. The performance of the GWO-ELM approach outperformed the NN, ELM, SVM (linear kernel), SVM (precomputed kernel), and RF in all experiments. This discovery confirms that generating the appropriate weights and biases for the ELM's single hidden layer decreases classification errors. In other words, avoiding inappropriate weights and biases prevents the ELM algorithm from becoming stuck in the local maxima of the weights and biases. Consequently, the performances of the proposed GWO-ELM approach in the multi-class and binary classification were impressive and achieved an accuracy of 96.21, 99.47, 96.15, and 99.04% using APTOS-2019 and IDRiD datasets, respectively. This research confirms that the combination of the GWO-ELM classifier with HOG-PCA features is an effective approach for detecting the DR using retinal images which could help physicians in easily screening for DR.

Furthermore, the proposed GWO-ELM technique is compared with some recent works (47–65) in terms of accuracy based on binary and multi-class classifications using APTOS-2019 and IDRiD datasets. Table 10 exhibits the comparison accuracy results of the proposed GWO-ELM and some other previous works.

**Table 10:** The comparison of accuracy between the proposed GWO-ELM and other previous works.

**Accuracy results based on APTOS-2019 dataset with 5 classes**		**Accuracy results based on APTOS-2019 dataset with 2 classes**	
**Method**	**Accuracy**	**Method**	**Accuracy**
DNN (50)	81.70	DNN (50)	97.41
Hybrid model (56)	86.34	DNN (51)	98.00
DNN (51)	82.54	Hybrid CNN-SVD and ELM (57)	99.32
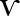 -SVM (52)	77.90	Ensemble (trimmed mean) (61)	98.60
MLP (55)	83.09	ResNet34 (47)	96.35
CNN512 (48)	89.00	CNN (62)	91.00
Tuned XGBoot (59)	94.20	RA-EfficientNet (64)	98.36
Proposed GWO-ELM	96.21	Proposed GWO-ELM	99.47
**Accuracy results based on IDRiD dataset with 5 classes**		**Accuracy results based on IDRiD dataset with 2 classes**	
**Method**	**Accuracy**	**Method**	**Accuracy**
MLP (53)	92.01	MLP (53)	98.87
ResNet50 + J48 (54)	92.46	CNN (58)	90.29
XG-Boost (49)	88.20	Coarse Network (63)	80.00
Lesion(Semi + Adv) (65)	91.34	HE-CNN (60)	96.76
Proposed GWO-ELM	96.15	Proposed GWO-ELM	99.04

Based on all the results in Table 10, it is clear that the performance of the GWO-ELM outperformed all the other previous works in binary and multi-class classifications using both datasets APTOS-2019 and IDRiD. This suggests that the proposed GWO-ELM is a reliable technique for the detection of DR when using image data. Although the proposed method has shown a good performance, there are some limitations which are provided as follows:

- The image datasets which have been used in this study for the training and testing purposes are small.
- The evaluations of this study did not consider the execution time measurement of the proposed GWO-ELM approach.
- The current study has considered only the off-line aspect for detecting DR.

## Conclusion

In this study, we have proposed a DR detection approach based on HOG-PCA features and GWO-ELM classifier. The GWO-ELM classifier underwent evaluations using the APTOS-2019 and IDRiD datasets. The outcomes indicated the superiority of the GWO-ELM over the existing methods [i.e., NN, ELM, SVM (linear kernel), SVM (precomputed kernel), and RF] (see Tables 6–10) in all experiments. In addition, the performance of the GWO-ELM classifier has been proven to outperform some recent studies (see Table 10) in both binary and multi-class classifications. The maximum multi-class classification performance of the GWO-ELM classifier was achieved with an accuracy reaching up to 96.21%. Further, the maximum binary classification performance of the GWO-ELM classifier was achieved with an accuracy of 99.47%. This demonstrates that the combination of the GWO-ELM and HOG-PCA is an effective classifier for detecting DR and might be applicable in solving other image data type. However, the current research has taken into account only the off-line aspect for detecting DR. Therefore, the future plan of the current research is to establish an approach to detect DR, which can handle the online execution for both classification and feature extraction in order to meet the real-time aspects. The proposed DR detection approach will be tested under adversarial attacks. Additionally, other optimization methods for ELM will be further explored in order to generate the most suitable weights and biases for the ELM which leads to minimizing classification process errors.

## Data availability statement

Publicly available datasets were analyzed in this study. This data can be found at: APTOS-2019: https://www.kaggle.com/competitions/aptos2019-blindness-detection/data; IDRiD: https://ieee-dataport.org/open-access/indian-diabetic-retinopathy-image-dataset-idrid.

## Author contributions

MAA: conceptualization, methodology, writing—original draft, software, writing review, and editing. MA: supervision, funding acquisition, and project administration. ST: supervision. FA-D: writing review and editing. MH: investigation. All authors contributed to the article and approved the submitted version.

## Funding

This work was supported in part by the Universiti Kebangsaan Malaysia under Grant DIP-2019-013.

## Conflict of interest

The authors declare that the research was conducted in the absence of any commercial or financial relationships that could be construed as a potential conflict of interest.

## Publisher's note

All claims expressed in this article are solely those of the authors and do not necessarily represent those of their affiliated organizations, or those of the publisher, the editors and the reviewers. Any product that may be evaluated in this article, or claim that may be made by its manufacturer, is not guaranteed or endorsed by the publisher.
